# Pembrolizumab Induced Calcitriol-Mediated Hypercalcemia

**DOI:** 10.1155/2023/9160326

**Published:** 2023-01-06

**Authors:** Tiffany T. Oommen, Kelly Sun, Javier Barranco-Trabi, Jeffrey Berenberg, Myungjin Kim

**Affiliations:** ^1^Tripler Army Medical Center, Department of Medicine, 1 Jarrett White Road, Honolulu, HI 96859, USA; ^2^Tripler Army Medical Center, Department of Hematology and Oncology, 1 Jarrett White Road, Honolulu, HI 96859, USA; ^3^Tripler Army Medical Center, Department of Nephrology, 1 Jarrett White Road, Honolulu, HI 96859, USA

## Abstract

PD-1/PD-L1 inhibitors such as pembrolizumab have radically improved the prognosis for many patients with advanced malignancies. Although revolutionary, its use can be complicated and limited by various immune-related adverse effects. Effective management depends on early recognition and prompt intervention. Herein, we describe a unique syndrome of hypercalcemia, with associated acute renal injury and hypoxic respiratory failure that was responsive to corticosteroids suggestive of immunotoxicity from pembrolizumab.

## 1. Introduction

Hypercalcemia in the setting of malignancy is relatively common and has been found to be driven by a variety of mechanisms. The major mechanisms by which hypercalcemia of malignancy can occur are osteolytic metastasis, humoral hypercalcemia with secretion of parathyroid hormone-related protein, and tumor production of calcitriol [1]. In normal circumstances, the conversion of 25-hydroxyvitamin D to 1,25-dihydroxyvitamin D occurs in the kidney under the physiologic regulation of PTH. Hypercalcemia should suppress PTH levels and in response, decrease production of 1,25-dihydroxyvitamin D. The lack of suppression of 1,25-dihydroxyvitamin D production points to extra renal production of 1,25-dihydroxyvitamin D independent from PTH from increased conversion of 25-hydroxyvitamin D by activated macrophages [2].

Pembrolizumab targets the PD-1/PD-L1 protein receptor and prevents the inhibition of T-cell activation [3]. Immunotherapy such as PD-1/PD-L1 inhibitors may play a role in abnormal macrophage activation and cause hypercalcemia in a rare subset of patients.

## 2. Case Presentation

A 63-year-old male with history of Stage IV undifferentiated malignant carcinoma, metastatic bladder cancer, and prostate cancer without known bone involvement on treatment with pembrolizumab for two years with recent addition of cisplatin/gemcitabine was presented. Two weeks following immunotherapy administration, he was hospitalized for fevers and malaise. He endorsed worsening shortness of breath and developed acute hypoxic respiratory failure. PET CT and CT pulmonary angiogram revealed left upper lobe airspace disease with hyper-metabolic activity, patchy ground glass opacities that is most prominent in the apices, and pulmonary emboli. Broad spectrum antibiotics were initiated in addition to treatment of pulmonary emboli with therapeutic dosage of low-molecular weight heparin.

**Figure 1:**
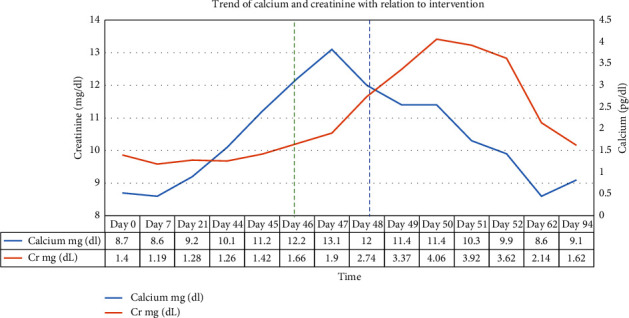
This graph shows the trend in the levels of calcium and creatinine throughout the patient's hospitalization as various treatments were provided as labeled above. “Day 0” is one day prior to pembrolizumab administration. “Day 94” represents post hospitalization recheck of laboratory studies. The *X* axis represents time measured in days. The left *Y* axis illustrates creatinine levels in mg/dL. The right *Y* axis demonstrates the calcium levels in mg/dL. The green dashed lines indicate the start dates for denosumab and the blue dash vertical dash lines indicate the start dates for methylprednisolone.

While admitted routine laboratory studies were significant for concurrent rising serum calcium to 13.1 mg/dl and serum creatinine of 2.80 mg/dL which peaked at 4.06 mg/dL (known baseline of 1.3-1.4 mg/dL), which is summarized in Figure 1. On urine sediment, there was a moderate amount of amorphous crystal-filled phosphorous which in the setting of alkaline urine with high serum calcium was presumed to be calcium phosphate. The worsening kidney function was thought to be driven by tubular injury from crystallopathy. Hypercalcemia was initially suspected to be related to significant volume depletion and malignancy, prompting treatment with intravenous normal saline, calcitonin 8 units/kg every 12 hours, and denosumab.

Further investigation revealed suppressed parathyroid hormone level of 7.1 pg/mL, parathyroid hormone-related protein (PTHrP) < 2.0 pmol/L, normal 25-hydroxy vitamin D level, elevated 1,25 dihydroxy vitamin D (calcitriol) of 143 pg/mL, and normal ACE. Despite 72 hours of aggressive treatment, his hypercalcemia was minimally responsive, as shown in Figure 1. His respiratory function continued to worsen requiring up to 5 liters per minute refractory to antibiotics and respiratory treatments. He was then started on methylprednisolone (2 mg/kg/day) for treatment of both this calcitriol-mediated hypercalcemia and pneumonitis. Within 24 hours of starting systemic steroids, calcium was down trending. By the time of discharge there was resolution of his hypoxic respiratory failure, hypercalcemia, and renal function. He was continued on daily prednisone upon discharge which was tapered over the course of four weeks. As an outpatient, there were no recurrent hypercalcemia and calcitriol levels stabilized. The calcitriol level obtained ten days following discharge was 77.8 pg/mL, repeat calcitriol level two months following discharge was 41.2 pg/mL; both within normal limits.

## 3. Discussion

This is a rare description of calcitriol-mediated hypercalcemia most likely related to PD-1/PD-L1 inhibitor use. There remains consideration that hypercalcemia from malignancy was a contributing factor; however, the refractory nature to temporizing therapies such as hydration and calcitonin and subsequent meaningful response to systemic steroid administration support pembrolizumab as the primary driver of our patient's hypercalcemia [1]. Additionally, denosumab was started two days prior to systemic steroids and may have contributed to long-term improvement; moreover, his calcium levels improved after steroid administration well before the median response time of denosumab [4].

Hypercalcemia in malignancy is often attributed to PTHrP or bone metastasis; however, sarcoid-like reactions causing hypercalcemia are described with use of PD-1/PD-L1 inhibitors. Granulomatous disease causes hypercalcemia by abnormal macrophage activation. The activity of ectopic 25(OH)D-1-hydroxylase (CYP27B1) expressed in macrophages leads to excessive 1,25(OH)2D formation [5]. Even without granulomatous features, it is postulated that PD-1/PD-L1 inhibitors can independently activate a subset of macrophages in cancer patients expressing PD-L1 [5–7]. This macrophage activation increases 25(OH) D-1-hydroxylase and shifts calcium homeostasis toward hypercalcemia [5]. The exact mechanism of macrophage activation is not well described. A study on the role of PD-1 on tumour-infiltrating macrophages in mice indicated that PD-1 plays a suppressive role in macrophages, inhibits macrophage phagocytosis, and may be associated with M2 polarization, while these effects may be reversed by anti-PD-1/PD-L1 antibody, with control of tumor progression [6]. This case supports this mechanism with elevated calcitriol, low PTH/PTHrP, and the absence of imaging findings typical for granulomatous disease or metastatic bone disease. On literature review, only one other isolated case has been previously described.

This case furthermore highlights a late-onset immune-related adverse event (irAE) occurring over two years since immunotherapy initiation. Classically, most reported irAEs occur soon after treatment initiation, typically within the first treatment cycle [3, 8]. Late onset irAEs continue to be underreported conditions that are difficult to predict or prevent [8]. There are ongoing discussions of appropriate surveillance strategies with no formal surveillance algorithms for late-onset irAEs to date [8].

The diagnosis and treatment of irAEs are highly dependent on individual clinicians to consider the heterogeneous presentations and broad-spectrum effect of checkpoint inhibitors. Holding a high index of suspicion can prompt a thorough evaluation and timely treatment of irAEs to minimize lasting end-organ dysfunction. In the case, the unique constellation of hypercalcemia and pneumonitis while undergoing treatment with immunotherapy should prompt consideration of an immune-related event.

Once diagnosed with an irAE, corticosteroids remain the mainstay of treatment for most PD-1/PD-L1 inhibitor immunotoxicities [3]. In this case, corticosteroids have a three-fold mechanism to restore calcium homeostasis by reversing the PD-1/PD-L1 inhibitor blockade, blocking 25(OH) D-1-hydroxylase and inhibiting osteoclast resorption [9]. Further investigation to characterize the effects of PD-1/PD-L1 inhibitors on calcium homeostasis is warranted to assist in timely identification and treatment of calcitriol-mediated hypercalcemia due to PD-1/PD-L1 inhibitors.

## Data Availability

No data were used to support this study.
